# Shear wave velocities in damaged kidneys: fast and slow

**DOI:** 10.1007/s00247-014-3070-5

**Published:** 2014-07-27

**Authors:** Øystein E. Olsen

**Affiliations:** Radiology Department, Great Ormond Street Hospital for Children NHS Foundation Trust, Great Ormond Street, London, WC1N 3JH UK

In this issue, Goya and coworkers [1] describe a well-performed study of sonographic elastography in relatively large groups of healthy children and children with varying degrees of renal damage as assessed by dimercaptosuccinic acid scintigraphy (DMSA). Their results are somewhat surprising: The more renal damage the slower the propagation of shear waves. This bewilders our intuitive belief that DMSA abnormality equals scarring equals fibrosis, fibrosis means stiffness, and stiffness should result in fast propagation of shear waves. It also conflicts with the report by Bruno et al. [2] that describes, concordant with expectations, significantly higher shear wave velocities in damaged as compared to non-damaged kidneys. The cause of the discrepancy may be multifactorial (inclusion criteria, measurement technique, etc.), and may not be easily resolved. However, several important lessons can be learned.

**Fig. 1:**
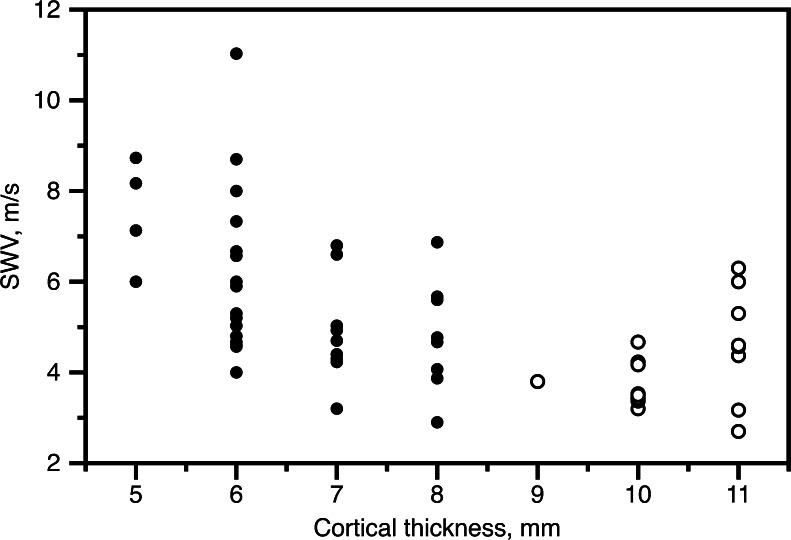
Shear wave velocity (SWV) of “affected” (black dots) and contralateral (circles) kidneys plotted against renal cortical thickness. Cortical thickness explains much of the variability in SWV. Adding information about renal damage does not improve the prediction of SWV and vice versa. Data re-analysed from Bruno et al. [2]

Bruno et al. [2] compared the “affected” and contralateral kidneys in children with unilateral renal damage. Along with measurements of shear wave velocities, they also measured and tabulated renal cortical thickness, renal length and renal pelvic diameter. But in their statistical model they only included “affected” as an explanatory variable, and indeed they found a significant effect. However, if we look critically at the numbers, a different story emerges. Figure 1 shows how shear wave velocities vary with renal cortical thickness in “affected” and contralateral kidneys. It is obvious that most of the variability in velocities can be explained by the differences in cortical thickness. In fact, once this is accounted for, there is no difference between the assumed damaged and non-damaged kidneys. (We do not need advanced statistical analysis here — simply rotate the page 20° or so counterclockwise.) In this sample, cortical thickness is a perfect determinant of renal damage -- just draw a vertical line through 8.5 mm to achieve 100% accuracy. Here the devil lies in the covariance of the two variables because if renal damages can simply be assessed by measuring the cortical thickness with US, then there is no need for the more involved and less accurate shear wave velocity measurement.

Unfortunately, Fig. 1 does not immediately help the interpretation of the unexpected observations made by Goya and coworkers, at least not if one expects that 1) renal damage seen on DMSA always corresponds to renal cortical thinning and 2) that a thinner cortex causes faster mechanical wave propagation. The latter assumption, based on Fig. 1, may not be possible to generalise. One could, for example, hypothesise that there are various degrees of fibrosis depending on the cause of damage and the time since damage, and that the parenchymal stiffness also depends on pressures within the renal collection system, etc. In other words, cortical thickness may not be an equally good determinant for shear wave velocity in other cohorts of patients. Extending this argument, “scarring” diagnosed by DMSA is unlikely to always mean fibrosis, so the intuitive sequence “DMSA scarring means fibrosis means stiffer parenchyma means faster wave propagation” is flawed.

The lessons learned: 1) Do not get taken by words —“DMSA scarring” may not only represent histopathological scarring, 2) Respect the data — convenient shortcuts may be invited by intuition, but at the risk of missing diagnostically important covariates, and 3) Significant relationships do not always imply added diagnostic value.

Goya et al. [1] offer an interesting insight into the complexity of diagnostic imaging. Their work should stimulate further investigation where the goals are improved early prediction of renal damage and less exposure of children to radionuclide modalities.
